# Gastrointestinal follicular lymphoma: using primary site as a predictor of survival

**DOI:** 10.1002/cam4.763

**Published:** 2016-10-01

**Authors:** Jay Chouhan, Sachin Batra, Rohan Gupta, Sushovan Guha

**Affiliations:** ^1^ Department of Internal MedicineThe University of Texas Health Science Center at HoustonHoustonTexas; ^2^ Department of GastroenterologyHepatology and NutritionThe University of Texas Health Science Center at HoustonHoustonTexas

**Keywords:** Epidemiology, gastrointestinal follicular lymphoma, primary site, prognostic factor, survival

## Abstract

Gastrointestinal follicular lymphoma (GI‐FL) is a rare extranodal variant of follicular lymphoma (FL) that has been increasingly reported in the literature. An especially indolent course is linked to the disease after a lack of observed patient death in past studies. However, overall survival (OS) and associated prognostic factors remain unclear. A large population‐based database was utilized to identify demographic and clinicopathologic characteristics of GI‐FL, along with survival differences among primary sites. The Surveillance, Epidemiology, and End Results Registry was used to identify GI‐FL cases between the years of 1973 and 2012. Kaplan–Meier curves compared OS differences and Cox proportional hazard models analyzed prognostic factors. Final analysis included 1109 cases. Small intestinal cases, which included those with single‐site and multi‐segment involvement, were most common (63.6%) followed by gastric (18.2%) and colorectal cases (18.2%). Small intestinal GI‐FL presented more frequently with grade I histology, and less often with grade III histology (*P* < 0.001 and *P* < 0.001, respectively). Small intestinal cases had better outcomes (5‐year OS = 80.9%, *P* < 0.001) compared to cases involving the stomach (5‐year OS = 52.7%) and colorectum (5‐year OS = 71.5%). On multivariate analysis for predictors of mortality, small intestinal involvement predicted for better survival; hazard ratio (HR) 0.66 (95% CI: 0.51–0.85). Advanced age (≥66), grade (grade III), and stage (Ann Arbor Stage III/IV) predicted for mortality with HR 5.46 (95% CI: 3.80–7.84), 1.42 (95% CI: 1.10–1.83), 1.57 (95% CI: 1.15–2.16), respectively. GI‐FL has poorer outcomes than previously suggested. Small intestinal involvement has a better prognosis. A possible biological basis for this will require further investigations in the future.

## Introduction

Follicular lymphoma (FL) is the second most common type of non‐Hodgkin lymphoma (NHL) in the United States, and makes up approximately 70% of all indolent NHL cases 1. The gastrointestinal (GI) tract is the most common extranodal presentation of primary NHL, and accounts for approximately 30–40% of such cases 2, 3. Gastrointestinal follicular lymphoma (GI‐FL) has been described as a rare disease that is estimated to account for only 1.0–6.0% of GI‐NHL cases 4, 5, 6, 7. However, after a description by Misdraji et al. in 1997, GI‐FL has been increasingly reported in the literature 6, 8, 9.

GI‐FL has been described as sharing the same immunophenotype, hallmark t(14;18)(q32;q21) translocation, and frequency of IgH/BCL2 rearrangements as nodal FL (N‐FL) 8, 10, 11, 12. However, evidence of unique clinical and biological characteristics have led GI‐FL to be considered as a separate variant of FL 6, 8, 10, 13, 14, 15, 16, 17, 18. The most notable of these traits has been a characteristic presentation in the small intestine as localized disease with grade I histology, which contrasts to the typically disseminated and higher grade‐presentation of N‐FL 8, 11, 13, 14, 15, 19, 20, 21. Additionally, GI‐FL has been described as possessing cellular and molecular characteristics not seen in cases of N‐FL, which instead show similarity to mucosa‐associated lymphoid tissue (MALT) lymphoma 17, 18, 22.

GI‐FL is linked to an excellent prognosis and a more indolent clinical course than N‐FL after previous studies have consistently lacked observed patient death 8, 15, 19, 20, 23. However, long‐term outcomes remain unclear, as past examinations have consisted of small cohort sizes and limited patient follow‐up 8. With no guidelines existing for the management of GI‐FL, understanding differences in survival among primary sites would likely aid clinical decision‐making. Thus, we reported the overall survival (OS) and associated prognostic factors of GI‐FL with a special emphasis on primary site through an analysis of a large population‐based database, the Surveillance, Epidemiology and End Results Registry (SEER).

## Methods

### Patients and methods

The SEER database was used to derive data regarding clinical features, treatment, and outcomes of patients diagnosed with GI‐FL from the years of 1974 through 2011. Data was derived from SEER 17 United States cancer registries participating in SEER program using SEER*STAT version 8.1.5 (NCI, Bethesda, MD).

GI‐FL cases were identified using the International Classification of Disease forOncology, Third Edition (ICD‐O‐3) histology codes categorizing them into grade 1 (9695/3), grade 2 (9691/3), grade 3 (9698/3), grade NOS (9690/3). GI primary site was identified using ICD‐O‐3 site codes C160‐C209. Case listings of GI‐FL patients were abstracted along with associated variables of interest, which included socio‐demographic features, histological grade and extent of disease, node positive disease, anatomical site, radiation therapy, and surgical intervention. Cases were excluded if patient age was <18 years and if details regarding the use of radiation or surgery were unknown. Anatomic primary sites for analysis included the stomach, small intestine, and colorectum. Specific SEER small intestinal subsite categories included multi‐site disease and single‐site involvement at the duodenum, jejunum, and ileum. Subsite categories were combined for analysis after studies investigating small intestinal GI‐FL with double balloon enteroscopy (DBE) or capsule endoscopy (CE) had evidenced ≥80% of the cases to have involvement at multiple subsites 15, 24, 25, 26. Disease stage was classified as localized (Ann Arbor stage I), regional (Ann Arbor stage II), and advanced (Ann Arbor stage III and IV). A fourth category of cases with unknown staging was also included for analysis. Node positivity is a category based on diagnosis of regional lymph nodes by a pathologist. Any case that had ≥1 regional lymph node diagnosed with disease was considered as node positive, cases with missing data regarding node positivity were categorized as unknown.

### Statistical analysis

SEER*Stat software (version 8.1.5, NCI) was utilized to calculate incidence rates from 1973 to 2010. Incidence data were age‐adjusted and normalized to the year 2000 US standard population.

All continuous data were summarized as mean and compared between anatomic locations using analysis of variance (ANOVA) and subsequent post‐hoc evaluation using Bonferroni multiple‐comparison test. Categorical data were reported as frequencies and compared using chi‐square or fisher's exact test, as appropriate. Kaplan–Meier time to event analysis and log‐rank tests were performed to calculate and compare survival between covariates of interest. Follow‐up time was calculated from the date of a patient's initial diagnosis until the date of last contact or date of death. Time to mortality was modeled as function of variables described above, using Cox proportional hazard models. All comparisons were considered significant when two‐sided *P* < 0.05. Missing values in categorical variables were treated as a level within the corresponding variable and included in the analysis to account for impact of missing data on association of other variables with mortality. All analyses were performed using *Stata Statistical Software: Release 13*; StataCorp LP, College Station, TX.

## Results

A total of 1109 GI‐FL cases were identified from the SEER database after applying the study's exclusion criteria. An age‐adjusted incidence rate of 4.3 patients per 100,000 was observed. The most common primary site was the small intestine (63.6%) followed by the stomach (18.2%) and colorectum (18.2%) as shown in Table 1. Among the small intestinal subsites, multi‐site involvement was most often present (24.1%) followed by single‐site occurrences at the duodenum (14.3%), ileum (13.8%), and jejunum (11.5%).

**Table 1:** Gastrointestinal follicular lymphoma primary site distribution

Primary site	Frequency (%)
Stomach	202 (18.2)
Small intestine	705 (63.6)
Duodenum	158 (14.2)
Jejunum	127 (11.5)
Ileum	153 (13.8)
Multi‐Site	267 (24.1)
Colorectum	202 (18.2)

Table 2 shows baseline characteristics and comparisons within GI‐FL primary sites categories. Males comprised 52.2% of the cohort with no significant difference in gender observed (*P* = 0.3). Significant differences in ethnicity (*P* = 0.001) and age (*P* < 0.001) were observed with white patients (80.9%) and those with ages ≥66 years (49.3%) seen with greatest frequency. Among primary sites, colorectal cases were more often diagnosed at a localized stage (Stage I, 45.5%) than gastric (21.3%) or small intestinal cases (31.6%, *P* < 0.001). Advanced histological disease (Grade III) was least often seen in the small intestine (11.1%) compared to the colorectum (13.9%) or stomach (33.2%, *P* < 0.001). Likewise, low‐grade histology (Grade I) was significantly more frequent (*P* < 0.001) among small intestinal cases (48.1%) compared to colorectal cases (39.6%) or gastric cases (19.3%). The majority of the cases lacked data regarding node positivity (66.8%). Of 1109 patients with GI‐FL, 64.7% of cases were treated with surgery while 11.8% were treated with radiation.

**Table 2:** Baseline characteristics and comparisons within primary site categories

Variable	Overall	Stomach	Small intestine	Colorectum	*P‐*value
	(*N* = 1109)	(*N* = 202)	(*N* = 705)	(*N* = 202)	
Sex					0.3
Male	579 (52.2)	101 (50.0)	363 (51.5)	115 (56.9)	
Female	530 (47.8)	101 (50.0)	342 (48.5)	87 (43.1)	
Age, y					<0.001
20‐50	168 (15.2)	20 (9.9)	117 (16.6)	31 (15.4)	
51‐65	394 (35.5)	54 (26.7)	268 (38.0)	72 (35.6)	
≥66	547 (49.3)	128 (63.4)	320 (45.4)	99 (49.0)	
Race					0.001
White	897 (80.9)	148 (73.3)	598 (84.8)	151 (74.8)	
Black	51 (4.6)	9 (4.5)	31 (4.4)	11 (5.5)	
Hispanic	75 (6.8)	23 (11.4)	31 (4.4)	21 (10.4)	
Asian/Pacific Islander	74 (6.7)	20 (9.9)	39 (5.5)	15 (7.4)	
Other	12 (1.1)	2 (1.0)	6 (0.9)	4 (2.0)	
Grade					<0.001
I	458 (41.3)	39 (19.3)	339 (48.1)	80 (39.6)	
II	206 (18.6)	36 (17.8)	137 (19.4)	33 (16.3)	
III	173 (15.6)	67 (33.2)	78 (11.1)	28 (13.9)	
Unknown	272 (24.5)	60 (29.7)	151 (21.4)	61 (30.2)	
Stage					<0.001
Stage I	358 (35.3)	43 (21.3)	223 (31.6)	92 (45.5)	
Stage II	200 (18.0)	14 (6.9)	154 (21.8)	32 (15.8)	
Stage III/IV	167 (15.1)	40 (19.8)	98 (13.9)	29 (14.4)	
Unknown	384 (34.6)	105 (52.0)	230 (32.6)	49 (24.3)	
Node Positive					0.02
Yes	94 (8.5)	25 (12.4)	59 (8.4)	10 (5.0)	
No	274 (24.7)	47 (23.3)	186 (26.4)	41 (20.3)	
Unknown	741 (66.8)	130 (64.4)	460 (65.3)	151 (74.8)	
Radiation					<0.001
Yes	131 (11.8)	40 (19.8)	68 (9.7)	23 (11.4)	
No	978 (88.2)	162 (80.2)	637 (90.4)	179 (88.6)	
Surgery					<0.001
Yes	718 (64.7)	92 (45.5)	488 (69.2)	138 (68.3)	
No	391 (35.3)	110 (54.5)	217 (30.8)	64 (31.7)	

N, number of patients; y, years.

Univariate Cox regression models (Table 3) showed that advanced age (age ≥66 years hazard ratio (HR) = 5.36, 95% CI: 3.75–7.66, *P* < 0.001), histological grade (grade III HR = 1.61, 95% CI: 1.27–2.05, *P* < 0.001) and stage (Stage III/IV stage HR = 1.43; 95% CI: 1.05–1.95; *P* = 0.02) were associated with mortality. Better survival was predicted by small intestinal primary site involvement (HR = 0.70, 95% CI: 0.55–0.89, *P* = 0.004). Factors associated with mortality on multivariate Cox regression models (Table 4) included older age, advanced stage, and histology. Longer outcomes were predicted by small intestinal involvement. Patients with ages between 51 and 65 years and ≥66 years were 2.1‐fold (HR: 2.11, 95% CI: 1.45 – 3.09; *P* < 0.001) and 5.5‐fold (HR: 5.46, 95% CI: 3.80–7.84, *P* < 0.001) more likely to die during follow‐up than patients with ages ≤50 years. Cases with advanced (Stage III/IV) and unknown stage were 1.6‐fold (HR: 1.58; 95% CI: 1.15–2.16; *P* = 0.005) and 1.4‐fold (HR: 1.46, 95% CI: 1.14–1.88, *P* = 0.003) more likely to die on follow‐up compared to cases with localized stage. Cases with grade III and unknown histology were similarly 1.4‐fold (HR: 1.42, 95% CI: 1.10–1.83, *P* = 0.007) and 1.4‐fold (HR: 1.39, 95% CI: 1.09–1.76, *P* = 0.008) more likely to pass during follow‐up than those with grade I disease. Conversely, small intestinal cases were 34% less likely to die on follow‐up than those with colorectal disease (HR: 0.66, 95% CI: 0.51–0.85, *P* = 0.001)).

**Table 3:** Univariate Cox proportional regression models for predictors of mortality

Variable	Hazard ratio (95% CI)		*P*‐value
Sex			
Male	1.16	(0.97–1.38)	0.1
Female	1	Reference	
Age, y			
20–50	1	Reference	
51–65	2.04	(1.40–2.97)	<0.001
≥66	5.36	(3.75–7.66)	<0.001
Race			
White	1	Reference	
Black	1.08	(0.71–1.63)	0.7
Hispanic	1.20	(0.86–1.69)	0.3
Asian/Pacific Islander	1.03	(0.72–1.47)	0.9
Other	0.81	(0.26–2.53)	0.7
Primary Site			
Stomach	1.25	(0.94–1.63)	0.1
Small Intestine	0.7	(0.55–0.89)	0.004
Colorectum	1	Reference	
Grade			
I	1	Reference	
II	1.30	(1.02–1.65)	0.04
III	1.61	(1.27–2.05)	<0.001
Unknown	1.41	(1.12–1.78)	0.004
Stage			
Stage I	1	Reference	
Stage II	0.82	(0.59–1.14)	0.2
Stage III/IV	1.43	(1.05–1.95)	0.02
Unknown	1.29	(1.01–1.63)	0.04
Node positive			
Yes	1.30	(0.95–1.79)	0.1
No	1	Reference	
Unknown	1.03	(0.85–1.27)	0.7
Radiation			
Yes	0.76	(0.58–0.98)	0.04
No	1	Reference	
Surgery			
Yes	0.92	(0.76–1.13)	0.5
No	1	Reference	

CI, confidence interval; y, years.

**Table 4:** Multivariate Cox proportional regression models for predictors of mortality

Variable	Hazard ratio (95% CI)		*P*‐value
Sex			
Male	1.19	(1.00–1.42)	0.05
Female	1	Reference	
Age, y			
20–50	1	Reference	
51–65	2.11	(1.45–3.09)	<0.001
≥66	5.46	(3.80–7.84)	<0.001
Race			
White	1	Reference	
Black	1.21	(0.80–1.83)	0.4
Hispanic	1.14	(0.80–1.61)	0.5
Asian/Pacific Islander	0.96	(0.67–1.38)	0.8
Other	0.88	(0.28–2.76)	0.8
Primary Site			
Stomach	0.91	(0.67–1.22)	0.5
Small Intestine	0.66	(0.51–0.85)	0.001
Colorectum	1	Reference	
Grade			
I	1	Reference	
II	1.23	(0.96–1.57)	0.10
III	1.42	(1.10–1.83)	0.007
Unknown	1.39	(1.09–1.76)	0.008
Stage			
Stage I	1	Reference	
Stage II	1.02	(0.73–1.44)	0.9
Stage III/IV	1.57	(1.15–2.16)	0.005
Unknown	1.46	(1.14–1.88)	0.003
Node positive			
Yes	1.15	(0.83–1.59)	0.4
No	1	Reference	
Unknown	0.95	(0.77–1.16)	0.6
Radiation			
Yes	0.88	(0.67–1.16)	0.4
No	1	Reference	
Surgery			
Yes	0.91	(0.74–1.12)	0.4
No	1	Reference	

CI, confidence interval; y, years.

Table 5 illustrated survival related to GI‐FL with significant differences in outcomes (*P* < 0.001) noted among primary sites of involvement. Small intestinal GI‐FL had longer outcomes (median OS = 165 months; 5‐year OS = 80.9% [95% CI: 77.7–83.7]; 10‐year OS = 62.7% [95% CI: 58.1–66.9]) compared to gastric GI‐FL (median OS = 68 months; 5‐year OS = 52.7% [95% CI: 45.4–59.4]; 10‐year OS = 40.0% [32.6–47.4]) or colorectal GI‐FL (median OS = 106 months; 5‐year OS = 71.5% [95% CI: 64.5–77.4]; 10‐year OS = 46.4% [95% CI: 36.6–55.5]). Cases diagnosed with advanced stage (Stage III/IV) had the worst outcomes (median OS = 129 months, 5‐year OS = 68.2% [95% CI: 60.3–74.9]; 10‐year OS = 54.4% [95% CI: 43.8–63.8]) among cases where staging information was available. Stage I had the highest median OS (median OS = 138 months) among all staging categories, but had a 5‐year OS (5‐Year OS = 79.0% [95% CI: 74.2–83.0]) and a 10‐year OS (10‐Year OS = 57.8% [95% CI: 49.6–65.1]) that was lower than stage II disease (5‐year OS = 79.8% [95% CI: 73.3–84.9]; 10‐year OS = 69.5% [95% CI: 60.7–76.7]). Cases with grade I histology (median OS = 167 months; 5‐year OS = 80.4% [95% CI: 76.4–83.9]; 10‐year OS = 63.9% [95% CI: 58.4–68.9]) had significantly longer outcomes (*P* < 0.001) than cases of any other grade category. Patients with ages ≥66 years had statistically worse outcomes (*P* < 0.001) compared to any other age group (median OS = 89 months, 5‐year OS = 59.9% [95% CI: 55.6–64.0], 10‐year OS = 39.1% [95% CI: 34.0–44.0]). No significant differences in outcomes were observed among cases diagnosed with nodal positivity (*P* = 0.3) or those receiving surgery (*P* = 0.5). However, significantly longer outcomes (*P* = 0.04) were observed among cases treated with radiation (median OS = 195 months; 5‐year OS = 80.7% [95% CI: 72.8–86.5]; 10‐year OS = 64.7% [95% CI: 54.7–73.0]).

**Table 5:** Survival of gastrointestinal follicular lymphoma

Variable	Median OS, m	5‐Year OS (95% CI)	10‐Year OS (95% CI)	*P*‐value
Age, y				<0.001
20–50	327	90.9 (85.1–94.6)	82.0 (73.7–87.9)	
51–65	204	86.7 (82.9–89.8)	68.8 (62.6–74.2)	
>66	89	59.9 (55.6–64.0)	39.1 (34.0–44.0)	
Primary site				<0.001
Stomach	68	52.7 (45.4–59.4)	40.0 (32.6–47.4)	
Small intestine	165	80.9 (77.7–83.7)	62.7 (58.1–66.9)	
Colorectum	106	71.5 (64.5–77.4)	46.4 (36.6–55.5)	
Grade				0.0003
I	167	80.4 (76.4–83.9)	63.9 (58.4–68.9)	
II	125	72.2 (65.4–77.9)	52.9 (44.3–60.7)	
III	102	64.3 (56.5–71.1)	45.3 (36.8–53.3)	
Unknown	115	70.7 (64.7–75.8)	49.7 (40.3–58.4)	
Stage				0.004
Stage I	138	79.0 (74.2–83.0)	57.8 (49.6–65.1)	
Stage II	a	79.8 (73.3–84.9)	69.5 (60.7–76.7)	
Stage III/IV	129	68.2 (60.3–74.9)	54.4 (43.8–63.8)	
Unknown	123	69.2 (64.3–73.6)	51.0 (45.8–56.0)	
Node positive				0.3
Yes	134	65.8 (55.2–74.4)	50.9 (39.5–61.2)	
No	129	76.2 (70.7–80.8)	56.6 (49.7–62.9)	
Unknown	145	74.1 (70.7–77.3)	56.2 (51.5–60.7)	
Surgery				0.5
Yes	138	75.0 (71.5–78.0)	56.3 (52.0–60.4)	
No	145	72.3 (67.3–76.6)	54.9 (47.8–61.4)	
Radiation				0.04
Yes	195	80.7 (72.8–86.5)	64.7 (54.7–73.0)	
No	136	73.1 (70.1–75.8)	54.4 (50.4–58.2)	

CI, confidence interval; m, months; OS, overall survival; y, years.

a Indicates that disease‐specific median OS was not reached.

Table 6 demonstrated the results of a separate survival analysis of localized GI‐FL cases (Stage I), diagnosed as having spread no farther than the GI primary site of origin. This was included, as some controversy exists over the definition of GI‐FL, specifically the ability to determine primary site of origin in cases with nodal involvement 5, 8, 27. Only cases diagnosed with grade I and grade II histology were included, as grade IIIB FL has been reported to have an aggressive clinical course similar to diffuse large B‐cell lymphoma 28. Among these cases, a significant difference in survival (*P* = 0.04) was noted with small intestinal GI‐FL having the longest outcomes (median OS = 144 months; 5‐year OS = 83.1% [95% CI: 75.5–88.5], 10‐year OS = 64.1% [95% CI: 51.5–74.3]) followed by colorectal GI‐FL (median OS = 124 months; 5‐year OS = 81.9% [95% CI: 66.9–90.6]; 10‐year OS = 55.8% [95% CI: 34.1–72.8]) and gastric GI‐FL (median OS = 51 months, 5‐year OS = 41.7% [95% CI: 10.0–70.8]; 10‐year OS = 41.7% [95% CI: 10.0–70.8]).

**Table 6:** Survival of localized stage (Stage I) and low‐grade (Grade I/II) gastrointestinal follicular lymphoma

Variable	*N*	Median OS, m	5‐Year OS (95% CI)	10‐Year OS (95% CI)	*P‐*value
Primary site					0.04
Stomach	9	51	41.7 (10.0–70.8)	41.7 (10.0–70.8)	
Small intestine	147	144	83.1 (75.5–88.5)	64.1 (51.5–74.3)	
Colorectum	47	124	81.9 (66.9–90.6)	55.8 (34.1–72.8)	

CI, confidence interval; m, months; N, number of patients; OS, overall survival.

## Discussion

GI‐FL is a rare subtype of GI‐NHL that has been increasingly reported in the literature. The disease has been associated with a lack of patient death and an especially indolent course; however, OS and prognostic factors remain unclear. This study represents the largest investigation of GI‐FL to date and is the first to utilize a population‐based database for analysis. Through our study, GI‐FL was observed as being more frequently diagnosed among Caucasian patients (80.9%) and those with age ≥66 years (49.3%). Similar to previous examinations, GI‐FL was most commonly diagnosed with small intestinal involvement (63.6%) and grade I histology (41.3%) when histological data were available. Among primary sites, cases of small intestinal GI‐FL had a significantly longer survival (5‐year OS = 80.9%, *P* < 0.001) compared to those involving the stomach (5‐year OS = 52.7%) or colorectum (5‐year OS = 71.5%). Through multivariate analysis, small intestinal involvement was found to independently predict longer outcomes (*P* = 0.001). Together, these results characterize GI‐FL as having shorter outcomes than previously suggested by the literature and indicate a potential prognostic role for small intestinal involvement in the future.

Previous studies have characterized GI‐FL as showing predilection for small intestinal involvement with 64% to 94% of previous populations diagnosed with related disease 15, 20, 23, 29, 30. Duodenal GI‐FL has made up the majority of these cases and has comprised 38% to 89% of previous study cohorts 13, 15, 20, 23, 29, 30. However, recent examinations utilizing more comprehensive small bowel DBE and CE for investigation have demonstrated small intestinal GI‐FL to have a predominantly multifocal nature with involvement of multiple subsites. These studies have evidenced involvement beyond the duodenum in 80–85% of small intestinal cases and additional jejunoileal involvement in 73–85% of cases previously diagnosed with duodenal disease 15, 24, 25, 26. Similar to the literature's descriptions, small intestinal GI‐FL made up the majority of our cohort (63.5%). However, we were limited in the ability to characterize overall disease involvement at each subsite secondary to SEER's lack of segmental delineation in multi‐segment cases.

GI‐FL has been characterized as having an excellent prognosis and a more indolent clinical course than N‐FL. This has come after a consistent lack of observed patient death in previous studies of GI‐FL, which was reflected in a review conducted by Yamamoto et al. that only found eight reported patient deaths out of 193 GI‐FL cases 8. Similarly, in a multicenter retrospective study that was one of the largest single investigations of GI‐FL in the literature, Takata et al. reported a 100% 5‐Year OS among 125 patients with stage I and II GI‐FL 15. Although no direct comparison can be made to prior studies of GI‐FL, we observed the survival at each of our study's primary sites to appear significantly lower than past characterizations of GI‐FL. Conversely, the 5‐Year OS of our cases that involved the small intestine (5‐Year OS = 80.9%) and colorectum (5‐Year OS = 71.5%) appeared comparable to previously reported outcomes of N‐FL (5‐Year OS = X‐X%). The gastric GI‐FL cases of our study, however, were noted to have an especially poor survival (5‐Year OS = 52.7%) 21, 31, 32.

Because GI‐FL is subject to varying interpretations in the literature, we performed a separate survival analysis of cases isolated to the GI tract (Table 6) 5, 8, 27. However, the outcomes from this analysis appeared similar to our overall cohort and lower than past GI‐FL studies 8, 15, 19, 20, 23, 29. Potential reasons for the difference in outcomes may lie in dietary, environmental, or genetic factors, as the majority of GI‐FL reports have come from Asia, specifically Japan and in patients of younger ages 8, 14, 15, 33, 34, 35. Routine use of upper endoscopy in those countries may have also played a role 6, 15. However, a more probable explanation is the relatively short 24.5‐month median follow‐up time of previous examinations. Additionally, the smaller populations of those studies may have created an aspect of reporting bias with respect to positive outcomes.

A precedent for lymphoma primary site, serving prognostic significance was made in cases of cutaneous B‐Cell lymphoma with leg involvement, which were initially found to have worse outcomes than other primary sites before a biological basis was discovered to account for the differences 36, 37, 38, 39. In our study, small intestinal GI‐FL cases had significantly longer outcomes (*P* < 0.001) than gastric or colorectal diseases, while involvement independently predicted for survival through multivariate analysis. This may indicate a potential prognostic role for small intestinal involvement in the future. If correct, explanation may come from past GI‐FL examinations that have focused on the duodenal segment and have described cellular and molecular characteristics similar to those seen in cases of MALT lymphoma 6, 14, 16, 17, 18. These similarities have included the usage of immunoglobulin heavy chains, expression of activation‐induced cytidine deaminase, and lack of the follicular dendritic cell meshworks commonly seen among cases of N‐FL 6, 17, 18, 19. More recently, GI‐FL has been evidenced as having a gene expression profile more similar to MALT lymphoma than N‐FL 22.

Although current guidelines are not available for the management of GI‐FL, some reports have suggested a “watch‐and‐wait” approach as an appropriate initial management strategy for asymptomatic patients with low‐ grade, low‐stage disease 19, 23, 29, 40. No definitive management recommendations can be made from this study secondary to the retrospective nature, and because radiation and surgery lacked prognostic significance in our analysis. However, we feel a more aggressive management approach may be warranted for future cases given the lower survival observed in our cohort in addition to reports that have described cases of GI‐FL showing an excellent response to various management strategies 8, 23.

There are limitations with the use of the SEER database specific to this study. Important prognostic factors regarding FL, specifically two of the four categories (Hemoglobin and LDH) of the Follicular Lymphoma International Prognostic Index (FLIPI) were not available for analysis. Having this data may have caused primary site, age, extent of disease, and histology to lack prognostic significance. However, FLIPI has been suggested to not confer prognostic significance in cases of GI‐FL and a study by Kiess et al. found that FLIPI did not predict outcomes among 40 patients with Stage I and II GI‐FL 19, 23.

This study also contains limitations shared by all examinations utilizing the SEER database. One limitation includes data that is based on coding from medical records with no accessible pathological data to confirm a diagnosis. However, there is a reported 93% consensus agreement in the diagnosis of FL, which is the highest among NHL subtypes 21. SEER is additionally limited by cases that are missing in categorical information. Because this study included such cases, notably those with missing data regarding disease stage and histologic grade, selective or variable reporting may have biased our survival analysis. The SEER database also lacks important treatment information regarding radiotherapy such as treatment dose, and whether therapy may be neoadjuvant or adjuvant in nature. Information is also lacking regarding chemotherapy and immunotherapy, both considered important treatment modalities in the management of FL.

Although the SEER database has limitations, it allowed for examination of long‐term outcomes associated with a rare tumor. Currently, this study represents the largest examination of GI‐FL to date. With the relative rarity of GI‐FL, we feel that it would have been difficult for any single or multi‐center study to reproduce our examination's results. SEER also allowed for GI‐FL to be characterized among a cohort that reflected the United States population. We feel this is important given that NHL has been shown to have a variable distribution around the world, and because the majority of reported GI‐FL cases have come from Japan where it has been suggested to be more prevalent 8, 14, 33.

In conclusion, GI‐FL has shorter outcomes than previously suggested. Primary site may serve as a potential tool for identifying cases with longer outcomes and guiding clinical decisions. In our study, small intestinal GI‐FL cases had longer outcomes than those of other primary sites with related involvement independently predicting for survival through multivariate analysis. The potential predictive value of small intestinal involvement may be explained by distinct cellular and molecular characteristics as evidenced by prior studies with further investigations still needed in the future.

## Conflict of Interest

No conflicts of interest exist.
